# Comparative Analysis of the Fecal Microbiota of Wild and Captive Beal’s Eyed Turtle (*Sacalia bealei*) by 16S rRNA Gene Sequencing

**DOI:** 10.3389/fmicb.2020.570890

**Published:** 2020-11-06

**Authors:** Jonathan J. Fong, Yik-Hei Sung, Li Ding

**Affiliations:** ^1^Science Unit, Lingnan University, Hong Kong, China; ^2^Ministry of Education Key Laboratory for Ecology of Tropical Islands, College of Life Sciences, Hainan Normal University, Haikou, China

**Keywords:** *Cetobacterium*, *Citrobacter*, gut microbiota, Hong Kong, 16S rRNA gene sequencing

## Abstract

The Beal’s eyed turtle (*Sacalia bealei*) is threatened with extinction due to hunting for large-scale trade. In Hong Kong, there are some of the world’s remaining wild populations of *S. bealei*, as well as a breeding colony. This breeding colony is at the core of conservation efforts (captive breeding, reintroduction programs). Therefore, we would like to know how captivity, in particular diet, affects the gut microbiota. Using high-throughput 16S rRNA gene sequencing, we comparatively analyzed the fecal microbiota of wild and captive *S. bealei*. We found that wild *S. bealei* have higher alpha diversity than captive *S. bealei*, but the difference was not significant. Significant differences were found in β-diversity; at the phylum level, wild *S. bealei* have higher relative abundances of Proteobacteria and captive *S. bealei* have higher relative abundances of Firmicutes. At the genus level, *Cetobacterium* and *Citrobacter* are more abundant in wild *S. bealei*, while *Clostridium* spp. are significantly more abundant in captive *S. bealei*. These results suggest conditions in captivity, with diet being a major factor, influence the gut microbiota of *S. bealei*. The connection between diet and health has always been considered for captive animals, and in this study we use the gut microbiota as an another tool to assess health.

## Introduction

The gut microbiota is known to influence a suite of host characteristics (nutrient acquisition, physiology, immunity, behavior, reproduction) (Fraune and Bosch, 2010; Columbo et al., 2015; Colston and Jackson, 2016), while the host diet and evolutionary history can influence the gut microbiota (Ley et al., 2009; Sanders et al., 2013; Clements et al., 2014). Conditions in captivity, such as diet and abiotic factors, are known to alter gut microbial diversity, with wild individuals tending to have a more diverse gut microbiota (Sonnenburg et al., 2016; Tan et al., 2018; Gao et al., 2019). This pattern has been demonstrated in the major vertebrate taxa: mammals (Gao et al., 2019), birds (Wang et al., 2017), reptiles (Keenan et al., 2013), and fish (Ramírez and Romero, 2017; Lv et al., 2018; Tan et al., 2018). Data from captive animals are poor predictors of wild microbiomes (Amato, 2013; Colston and Jackson, 2016), underpinning the importance of studying wild microbiomes (Hird, 2017).

Gut microbiotas of non-mammal vertebrates are poorly studied (Colston and Jackson, 2016); most vertebrate gut microbiota studies focus on mammals (human, mouse, rat) and model organisms (zebrafish). Turtles are particularly poorly represented in the gut microbiota literature, but the available studies suggest the gut microbiota is influenced by diet, geography, and ontogeny. For diet, turtle groups with different ecologies (sea turtles, freshwater turtles, tortoises) show a similar trend; species with plant-based diets had gut microbiota dominated by the bacterial phylum Firmicutes (Gaillard, 2014; Modica, 2016; Fugate et al., 2019; Peng et al., 2020), while species with animal-based diets were dominated by Bacterioidetes (Biagi et al., 2018; Bloodgood et al., 2020). In this study, we focus on the freshwater Beal’s eyed turtle (*Sacalia bealei*) from southern China (Anhui, Fujian, Guangdong, Guangxi, Guizhou, and Jiangxi Provinces, and Hong Kong) (Shi et al., 2008). This species, along with a majority of other Asian turtle species, is threatened with extinction due to their large-scale trade for food, medicine, and as pets (van Dijk et al., 2000). In Hong Kong, some of the world’s remaining wild populations of *S. bealei* exist, as well as a captive breeding colony to support *ex situ* conservation of this species. Captive breeding in Hong Kong has been unsuccessful for the past seven years (HKHerp, pers. comm.), and we deduced that some conditions in captivity are suboptimal and contribute to these failures. One of the major differences between wild and captive individuals is their diet; captive *S. bealei* are fed commercial turtle food, of which the top two ingredients are soybean meal and wheat, while wild individuals have a more diverse diet dominated by fruits and terrestrial insects (Sung et al., 2020). We investigate the influence of a captive environment, in particular diet, by comparing the gut microbiotas of captive and wild *S. bealei*.

To gain insight into the composition of gut microbiota in *S. bealei* under different food-source conditions, we used high-throughput 16S rRNA sequencing to characterize and compare the fecal microbiota of wild and captive *S. bealei*. In addition to providing the first description of the fecal microbial diversity of an Asian freshwater turtle, our results have immediate implications for the conservation of an endangered species. The microbiota analysis strategy (16S rRNA analysis) could serve as a scientific basis to improve the management of the captive breeding colony.

## Materials and Methods

### Sample Collection

Due to the endangered status and rarity of *S. bealei*, the number of individuals available to study is relatively low. We include eight turtles in this study, four wild *S. bealei* (WS) and four captive *S. bealei* (CS). The four wild individuals were caught from two sites in Hong Kong using baited aquatic traps. The naming of individuals reflects this, with the first number being site (either 1 or 2) and the second number being the individual (WS1.1,WS1.2, and WS1.3 from site 1 and WS2.1 from site 2). Due to the status of this species (Endangered on the IUCN Red List; protected in Hong Kong under Cap. 170—Wild Animals Protection Ordinance), the detailed locality information is not specified. We estimate the population size in Hong Kong to be less than 100 wild individuals. The diet of wild individuals is diverse and similar across the two sites, dominated by fruits and terrestrial insects (Sung et al., 2020). The four captive individuals are members of a breeding colony started in 2013 and maintained by HKHerp and Ocean Park Hong Kong (CS1.1, CS1.2, CS1.3, and CS1.4). The breeding colony consists of 40 individuals originating from the pet trade, all with unknown geographic origin. Turtles are kept in groups of 4–6 in individual aquaria, and fed commercial turtle food (Zoo Med Natural Aquatic Turtle Food-Growth Formula) occasionally supplemented with other items (e.g., mealworms and blueberries). General information (age class, sex, carapace length) of the eight individuals is in Supplementary Table S1. One individual was a juvenile of unknown sex (WS1.1), while there were five adult females (WS1.2, WS1.3, WS2.1, CS1.2, CS1.4) and two adult males (CS1.1, CS1.3).

Fecal samples have been shown to be a good proxy to estimate the microbial diversity of the distal gut (Videvall et al., 2017; Yan et al., 2019). Fecal samples were collected by placing individuals in a container with a wire mesh floor (Supplementary Figure S1). The excreted feces would fall through the wire mesh floor, preventing the individual from stepping on and contaminating the sample. Feces for each individual were immediately collected in a sterile 2 mL tube and frozen at -80°C. After sample collection, the apparatus was sterilized with a 10% bleach solution.

### DNA Extraction and PCR Amplification

Total DNA of fecal samples was extracted using E.Z.N.A.^®^ Soil DNA Kit (Omega Bio-Tek; Norcross, Georgia, United States). The concentration and purification of DNA were measured using a NanoDrop 2000 (Thermo Fisher Scientific; Wilmington, United States). The V3-V4 hypervariable region of the bacterial 16S rRNA gene was amplified with primers 338F (5′- ACTCCTACGGGAGGCAGCAG-3′) and 806R (5′-GGACTACHVGGGTWTCTAAT-3′). PCR reactions for each sample were performed in triplicate in 20 μl reactions containing 4 μl of 5× FastPfu Buffer, 2 μl of 2.5 mM dNTPs, 0.8 μl of each primer (5 μM), 0.4 μl of FastPfu polymerase and 10 ng of template DNA. The following thermal cycler program was used for amplification: 3 min at 95°C, 27 cycles of 30 s at 95°C, 30 s at 55°C, and 45 s at 72°C, and a final extension at 72°C for 10 min. The PCR products were purified using the AxyPrep DNA Gel Extraction Kit (Axygen Biosciences; Union City, California, United States) and quantified using a QuantiFluor^TM^-ST (Promega; Madison, Wisconsin, United States).

### High-Throughput Sequencing and Data Processing

Purified amplicons were pooled in equimolar concentrations (11 ng DNA for each sample) and paired-end sequenced (2 × 300) on an Illumina MiSeq platform (Illumina; San Diego, California, United States) according the standard protocols of Majorbio Bio-pharm Co., Ltd. (Shanghai, China). The raw reads were submitted to the NCBI Sequence Read Archive (SRA) database (accession number: PRJNA623155).

Raw FASTQ file reads were quality-filtered with Trimmomatic (Bolger et al., 2014) by truncating reads at any site receiving an average quality score <20 over a 50 bp sliding window and removing reads if they contained ambiguous bases or primer sites had >2 nucleotide mismatches. Reads were then merged with FLASH (Magoè and Salzberg, 2011) if they had matching overlap longer than 10 bp. All samples were rarefied to the sample with the lowest number of reads. Operational taxonomic units (OTUs) were clustered with a threshold of 97% similarity cutoff using UPARSE v.7.1 (Edgar, 2013) and chimeric sequences were identified and removed using UCHIME (Knight, 2011). All singleton OTUs were removed from the dataset. Bacterial taxonomy was assigned to the species level using the SILVA database (Release 138.1)^1^, removing non-relevant OTUs (eukaryote, mitochondria, chloroplast, unclassified).

### Alpha and Beta Diversity Analyses

Rarefaction curves were created in Mothur v.1.30.1 (Schloss et al., 2009) to determine whether sequencing depth was sufficient to cover the expected number of OTUs at the level of 97% sequence similarity. Alpha diversity indices (i.e., ACE, Chao1, Shannon, Simpson) were measured from the rarefied OTU dataset in Mothur (Caporaso et al., 2010) for richness and diversity of bacterial community. The Kolmogorov-Smironov (K-S) test and Homogeneity Variance (H-V) test were used to test whether the data were normally distributed and homogenous, respectively, using IBM SPSS Statistics 22. If the data were normally distributed and homogenous, a Student’s *t*-test was used to evaluate whether alpha diversity indices were statistically significant, while a Wilcoxon rank-sum test was used otherwise. Beta diversity was visualized by principal coordinate analysis (PCoA), based on unweighted UniFrac distances analyses. Analysis of Similarities (ANOSIM) was performed to determine the differences among groups (Clarke, 1993) using the Bray–Curtis similarity index as a metric of similarity between the bacterial communities based on the abundance of OTUs between samples. PCoA, Venn diagrams, and ANOSIM were produced using R (R Core Team, 2020).

### Bacteria Composition and Relative Abundance

Community structure was analyzed at the phylum, family, and genus levels. Relative abundances are presented as means ± SD. The significant difference between WS and CS were calculated using either a Student’s *t*-test or Wilcoxon rank-sum test depending on the distribution of the data. A *P*-value < 0.05 was considered to be statistically significant. Additional analyses were performed to infer the species of candidate genera, genera showing significant differences between WS and CS. We first compared the sequences to the GenBank database using BLAST, followed by constructing a phylogenetic tree. There are limitations to this approach (low phylogenetic resolution due to the short DNA fragment, misidentifications in GenBank), but we proceed to infer the potential function of the bacterial taxa. The new sequences were aligned using MUSCLE (Edgar, 2004), to a maximum of five top BLAST hits with identification to the species or genus level. The phylogenetic tree was inferred using maximum likelihood in RAxML v.8.2.4 (Stamatakis, 2014), using the combined rapid bootstrap and search for the best-scoring tree, 1000 bootstrap replicates and the GTR + G model of nucleotide substitution. The revised identification is based on the results of BLAST and the phylogenetic tree.

Linear discriminant analysis effect size (LEfSe) was performed to determine the taxa most likely to explain the differences between WS and CS, using the LEfSe software, with the filter value of the LDA score set as 4 by default (Segata et al., 2011).

## Results

### Analysis of rRNA Sequencing Results

The number of quality-filtered sequences obtained for each sample was 32,115–71,094, for a total of 432,258 sequences (213,840 reads from WS and 218,418 reads from CS). The length distribution was 411–423 bp, with an average length of 416 bp (Supplementary Table S1 and Supplementary Figure S2). All samples were rarefied to 32,115 reads (sample CS1). All rarefaction curves reached the saturation phase (Supplementary Figure S3), indicating that sufficient sampling depth was achieved for each sample.

### Alpha and Beta Diversity Analyses

The four alpha diversity indices (Shannon, Simpson, ACE, Chao1) of WS and CS are displayed in Table 1. All K-S and H-V tests were not significant (*P* > 0.05), indicating that these data were normally distributed and homogenous, so Student’s *t*-test was used for tests of statistical significance. WS had higher values for OTU, Shannon, ACE and Chao1, while CS had a higher value for Simpson. In summary, all indices pointed toward WS having higher diversity than CS, but none of these differences were significant (*P* > 0.05).

**TABLE 1:** Alpha diversity of gut microbiota in fecal samples from wild (WS) and captive (CS) *Sacalia bealei*.

Alpha diversity	WS	CS	*P*-value (Kolmogorov-Smironov test)	*P*-value (Homogeneity-Variance test)	*P*-value (Student’s *t*-test)
Shannon	3.00 ± 0.87	2.70 ± 0.75	0.20	0.68	0.618
Simpson	0.12 ± 0.07	0.15 ± 0.09	0.11	0.42	0.583
ACE	235.65 ± 124.77	224.99 ± 41.94	0.20	0.22	0.877
Chao1	231.93 ± 125.15	215.91 ± 55.44	0.20	0.31	0.823
OTU	213 ± 134.39	194.25 ± 58.19	0.20	0.25	0.807

*Data are mean ± SD. Student’s t-test were used to test for significance.*

**FIGURE 1:**
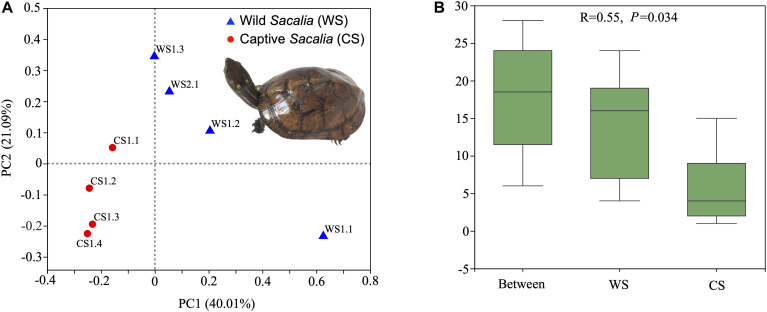
Beta diversity and ANOSIM analyses. **(A)** Principal Coordinates Analysis (PCoA) plot of beta diversity based on unweighted UniFrac distances for bacterial communities in fecal samples of eight Beal’s eyed turtles (*Sacalia bealei*). The main coordinates (PC1 and PC2) are represented in the axes, and their relative contributions are denoted by the percentage in parentheses. **(B)** ANOSIM analysis. *R*-value range (–1, 1). An *R*-value close to 0 represents no significant differences in inter-group and intra-group. An *R*-value close to 1 shows that inter-group differences are greater than intra-group differences. The *P*-value represents the confidence level of the statistical analysis; *P* < 0.05 reflects a statistically significant difference. The y-axis represents the distance rank between samples, and the x-axis represents the results between both groups. Intra-group results are shown for each group.

Beta diversity analyses are illustrated as PCoA coordinate plots based on unweighted UniFrac distances (Figure 1A). Wild and captive individuals are separated in the ordination plot, indicating that the bacterial communities of WS and CS were different. ANOSIM analysis showed the different composition between wild and captive *S. bealei* (*R* = 0.55, *P* = 0.034). The inter-group differences in gut microbiota composition of WS and CS were greater than the intra-group differences, and the composition difference in the gut microbiota between both groups was significant (*P* < 0.05) (Figure 1B). Although samples WS1.1 and CS1.1 were statistically similar to their respective groups, they were spatially separated from other wild and captive samples, respectively, in the PCoA plot. The difference of WS1.1 compared to other wild individuals may be due to differences in age class or sex [WS1.1 juvenile (sex unknown), others adult females], while there was no obvious difference (age class, sex, size) between CS1.1 and other captive samples.

### Bacteria Composition and Relative Abundance

**FIGURE 2:**
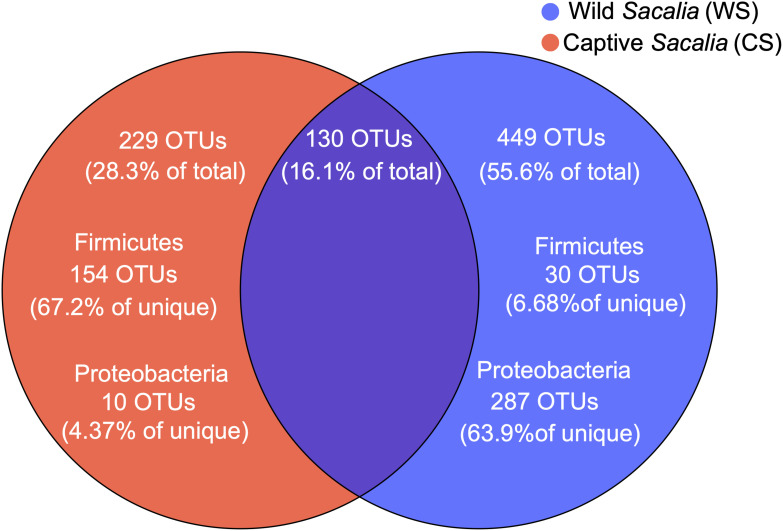
Venn diagram showing the unique and shared OTUs in wild (WS, blue) and captive (CS, red) *Sacalia bealei*. From the unique OTUs, a majority were Firmicutes (71.6%) for CS and a majority were Proteobacteria (63.9%) for WS.

The shared and unique microbiota of wild and captive *S. bealei* are displayed using a Venn diagram (Figure 2). Among the total 808 OTUs, 130 OTUs (16.1%) were shared between groups, with 75 OTUs belonging to the phylum Firmicutes (57.7% of shared), 27 OTUs belonging to Proteobacteria (20.8% of shared), and 13 OTUs belonging to Bacteroidetes (10% of shared). For WS, 449 OTUs (55.6% of total) were unique, with most belonging to the phylum Proteobacteria (287 OTUs, 63.9% of unique), and a limited number belonging to Firmicutes (30 OTUs, 6.68% of unique). For CS, 229 OTUs (28.3% of total) were unique, with most belonging to Firmicutes (154 OTUs, 67.2% of unique), and a limited number belonging to Proteobacteria (10 OTUs, 4.37% of unique) (Figure 2).

**FIGURE 3:**
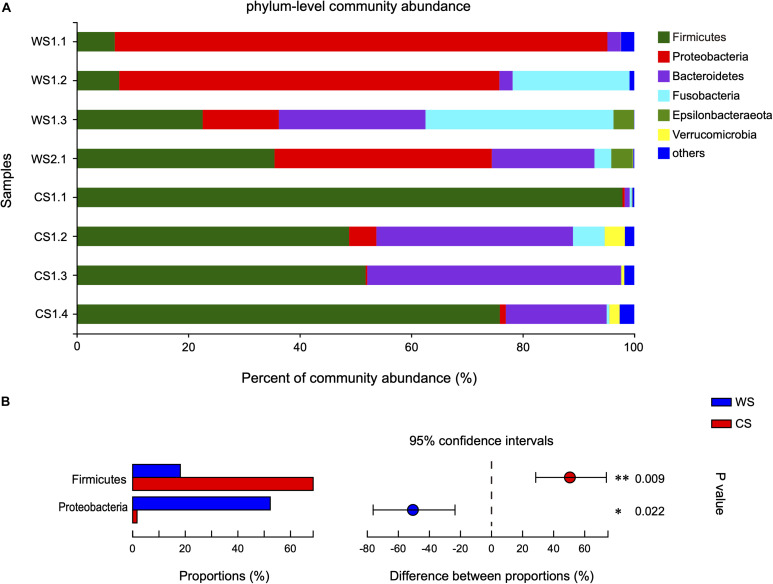
Comparison of bacterial community at the phylum level in wild and captive *Sacalia bealei*. **(A)** Barplots of community abundance. Taxa with abundances <2% have been combined under “others.” **(B)** The significant phylum in the abundances >2%. The phyla significantly different between groups are indicated (*P < 0.05, **P < 0.01). *P-*values are based on Wilcoxon rank-sum test or Student’s *t*-tests, depending on the distribution of the data. WS = Wild *Sacalia*, CS = Captive *Sacalia*.

The 808 OTUs were classified into 23 phyla, 35 classes, 93 orders, 165 families, 358 genera, and 511 species. At the phylum level, the community abundance of the most common taxa is shown in Figure 3A. The WS microbiota was more even, being dominated by Proteobacteria (52.3%), followed by Firmicutes (18.12%), Fusobacteria (14.42%), and Bacteroidetes (12.37%). In contrast for CS, Firmicutes (68.61%) and Bacteroidetes (24.94%) were the two most abundant phyla, accounting for 93.55% of total sequences. Proteobacteria (1.67%) and Fusobacteria (1.63%) were rare in CS. It should be noted that the proportion of Proteobacteria in sample WS1.1 was relatively higher (88.36 vs. 40.28%) than other WS samples, while the proportion of Firmicutes in sample CS1.1 was relatively higher (97.89 vs. 58.85%) and Bacteroidetes relatively lower (0.90 vs. 32.95%) than other CS samples. The relative abundances of Firmicutes and Proteobacteria were significantly different between WS and CS (Figure 3B and Supplementary Table S2). To assess the influence of WS1.1 and CS1.1 on the results, we removed these two samples from analyses, and Firmicutes was still statistically significant between groups (*p* = 0.035), while Proteobacteria was not (*p* = 0.073).

**FIGURE 4:**
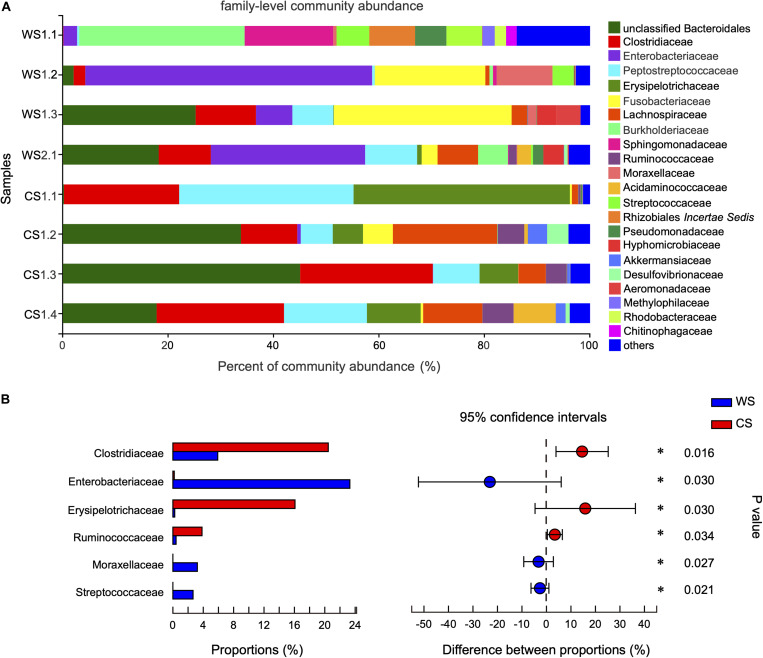
Comparison of community at the family level in wild and captive *Sacalia bealei*. **(A)** Barplot of community abundance. Taxa with abundances <2% have been combined under “others.” **(B)** The significant family in the abundances of top 15. The family significantly different between groups are indicated (*P < 0.05). *P-*values are based on Wilcoxon rank-sum test or Student’s *t*-tests, depending on the distribution of the data. WS = Wild *Sacalia*, CS = Captive *Sacalia*.

At the family level, the community abundance is shown in Figure 4A. The families significantly higher in WS compared to CS were Enterobacteriaceae (WS: 23.3%, CS: < 0.02%), Moraxellaceae (WS: 3.22%, CS: < 0.01%), and Streptococcaceae (WS: 2.66%, CS: 0.00%). The families significantly higher in CS compared to WS were Clostridiaceae (CS: 20.46%, WS: 5.91%), Erysipelotrichaceae (CS: 16.08%, WS: 0.24%), and Ruminococcaceae (CS: 3.84%, WS: 0.42%) (Figure 4B and Supplementary Table S3).

**FIGURE 5:**
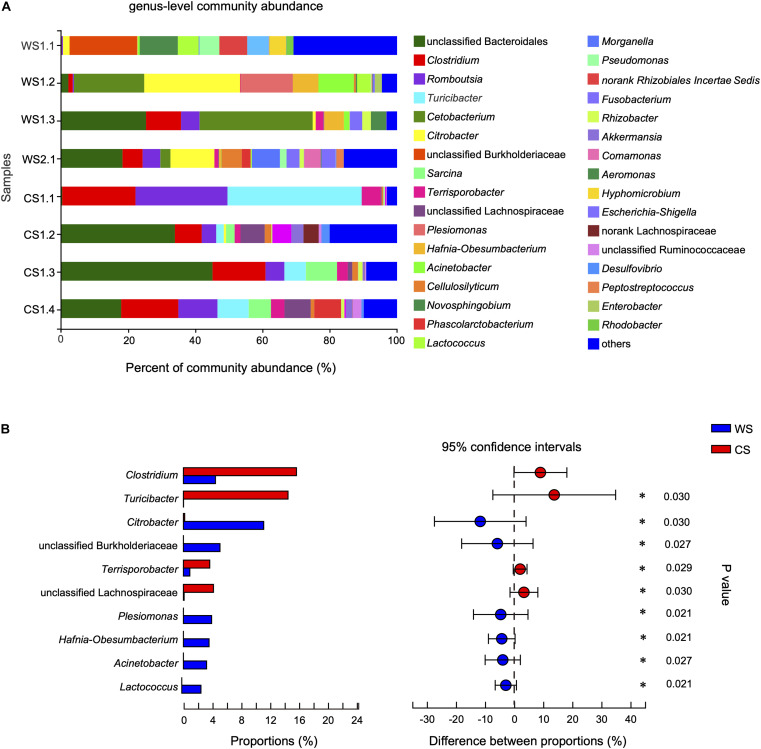
Comparison of community at the genus level in wild and captive *Sacalia bealei*. **(A)** Barplot of community abundance. Taxa with abundances <2% have been combined under “others.” **(B)** The significant genera in the abundance of top 20. Genera significantly different between groups are indicated (*P < 0.05). *P-*values are based on Wilcoxon rank-sum test or Student’s *t*-tests, depending on the distribution of the data. WS = Wild *Sacalia*, CS = Captive *Sacalia*.

At the genus level, the community abundance is shown in Figure 5A. The genera significantly higher in WS compared to CS were *Citrobacter*, unclassified Burkholderiaceae, *Plesiomonas*, *Hafnia-Obesumbacterium*, *Acinetobacter*, and *Lactococcus* (Figure 5B and Supplementary Table S4). In contrast, the genera significantly higher in CS compared to WS were *Clostridium*, *Turicibacter*, *Terrisporobacter*, and unclassified Lachnospiraceae (Figure 5B and Supplementary Table S4). In addition, the abundance of genus *Cetobacterium* in wild *S. bealei* (14.42%) was higher than in CS (< 0.01%), though the difference was not significant (Supplementary Table S4). A total of 70 OTUs were contained in these 10 candidate genera. When possible, the identification of these candidate genera was revised based on BLAST searches and phylogenetic analysis (Supplementary Figure S4 and Supplementary Table S5).

**FIGURE 6:**
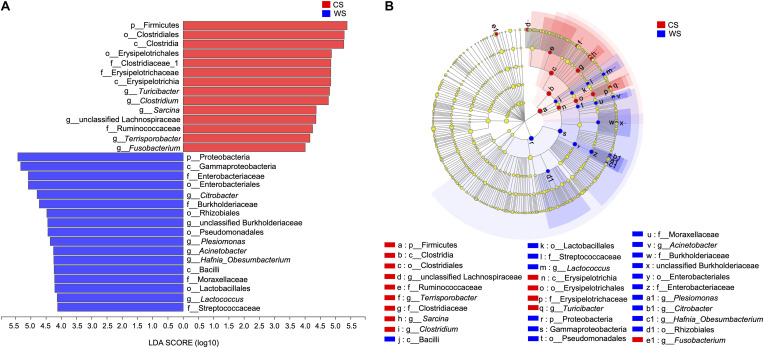
Linear discriminant analysis effect size (LEfSe) **(A)**, The bar graph of LDA scores showing the taxa with statistics difference between wild (WS) and captive (CS) *Sacalia*. The degree of influence of a species was expressed by the length of the bar. Only taxa meeting an LDA significant threshold >4 are shown. **(B)** The cladogram of taxa showing significant difference between groups. Red and green dots represent the core bacterial populations in WS and CS, respectively.

From the LEfSe analysis, 17 WS and 14 CS nested taxa were identified to explain the differences between groups (Figure 6). Generally, as seen in our other analyses, WS was characterized by taxa in phylum Proteobacteria, while CS was characterized by taxa in the phylum Firmicutes. Exceptions to this pattern are WS having significantly more OTUs in class Bacilli (phylum Firmicutes) and genus *Fusobacterium* (phylum Fusobacteria).

## Discussion

Previous studies have shown that diet is the main factor affecting the gut microbiota in mammals (Schwab et al., 2011; Gao et al., 2019). The diet of wild *S. bealei* consists mainly of fruits and terrestrial insects (Sung et al., 2020), while captive *S. bealei* are fed commercial turtle food, of which the top two ingredients are soybean meal and wheat. Thus, differences between microbiota of captive and wild *S. bealei* might be strongly associated with these dietary differences. Although our sample size was relatively large for endangered Asian turtles, the sample size in a statistical sense are small, and therefore findings considered tentative. The present study is the first to investigate the composition of gut microbiota in *S. bealei* and comparison between wild and captive individuals using high-throughput 16S rRNA sequencing technology.

### Comparison of Wild and Captive Individuals

Although alpha diversity analyses indicated no significant difference between wild and captive individuals (Table 1), beta diversity analyses show significant differences (Figure 1). The differences are driven primarily by the diversity and relative abundances of Proteobacteria (many unique to WS) and Firmicutes (many unique to CS) (Figures 2, 3, 6). The standard deviations for alpha diversity indices were relatively high, likely driven by differences in two samples (WS1.1 and CS1.1). However, statistical analyses (K-S and H-V tests) found that the data were normally distributed and homogenous, and providing some confidence in the results. Wild turtles in our study possess a more diverse (even and species rich) microbiota than captive individuals, a general pattern found across a variety of vertebrate taxa: mammals (Gao et al., 2019), birds (Wang et al., 2017), reptiles (Keenan et al., 2013), and fish (Ramírez and Romero, 2017; Lv et al., 2018; Tan et al., 2018).

A difference in the relative proportion of bacterial taxa can shed light on a dietary change. A trend first found in humans, individuals with plant-based diets have gut microbiota dominated by taxa in the phylum Firmicutes that metabolize plant polysaccharides and degrade cellulose into volatile fatty acids, while animal-based diets are dominated by taxa in the phyla Proteobacteria that are bile tolerant (David et al., 2014). This pattern was also seen in turtle studies, with herbivorous species having gut microbiota dominated by Firmicutes (Gaillard, 2014; Modica, 2016; Fugate et al., 2019; Peng et al., 2020), while carnivorous and omnivorous species had gut microbiotas dominated by Bacteroidetes (Biagi et al., 2018; Bloodgood et al., 2020). In particular, Bloodgood et al. (2020) showed that gut microbial diversity has the ability to change in relation to diet; when herbivorous green sea turtles (*Chelonia mydas*) were given an omnivorous diet (seafood + vegetables) in captivity, their gut microbiota shifted from being dominated by Firmicutes to Bacteriodetes. In our study, the proportion of Bacteroidetes was higher in captive *S. bealei* (12.37 ± 11.97% in WS, 24.94 ± 19.62% in CS), as well as Firmicutes (18.12 ± 13.65% in WS, 68.61 ± 22.97% in CS). Although both phyla increased in captive individuals, the larger proportional increase and higher number of unique Firmicutes points toward a larger proportion of plant-based foods in the captive diet. This is likely due to the commercial food given to captive individuals, of which the top two ingredients are soybean meal and wheat flour.

In addition, the proportions of Proteobacteria (1.67 ± 2.18% in CS, 52.3 ± 32.78% in WS) and Fusobacteria (1.63 ± 2.69% in CS, 14.42 ± 15.84% in WS) were higher in wild compared to captive individuals. These results were further supported by the LEfSe analysis, identifying Proteobacteria and *Fusobacterium* (phylum Fusobacteria) as characteristic taxa of WS. Most of the unique OTUs in wild *S. bealei* belong to Proteobacteria (Figures 2, 3). Proteobacteria taxa have a wide range of functions, including metabolizing compounds (carbohydrates, proteins, amino acids, cofactors), participating in respiration, and repairing proteins damaged by oxidation (Moon et al., 2018). We hypothesize that the high abundance of Proteobacteria provides wild *S. bealei* with a gut microbial community that is more resilient and adaptable to the changing environment in nature. This is in contrast to captive individuals, where conditions, such as food type and availability are more consistent. In our dataset, Fusobacteria is represented by two OTUs in from the family Fusobacteriaceeae identified as *Fusobacterium* (OTU231) and *Cetobacterium* (OTU320). *Cetobacterium*, which plays an important role in anaerobic metabolism (Larsen et al., 2014), is relatively abundant in the gut of some freshwater fish species and plays an important role in producing vitamin B-12 (Sugita et al., 1991; Tsuchiya et al., 2008). Whether the high abundance of *Cetobacterium* in wild *S. bealei* (14.42% vs. <0.01% in CS) plays a similar role in freshwater turtles needs further investigation.

It is worth noting that although the WS and CS data were normally distributed and homogenous (Table 1), the microbiota WS1.1 and CS1.1 were visually different compared to their respective groups (Figures 3–5). We explore these samples in detail here. WS1.1 had a higher proportion of the phylum Proteobacteria (88.36 vs. 40.28% in other samples). Since one of the main functions of Proteobacteria is protein metabolism, we deduce this individual ate a higher proportion of animal-based foods. This could be due to individual or age class variation, as this was the only juvenile individual WS (the other three were adult females). Since it is difficult to identify the sex of juveniles, it is unclear whether this difference is a between males and females. In a stable isotope study of *S. bealei* diet, adults and males had larger isotopic niche sizes, meaning they eat a higher diversity of prey items (Sung et al., 2020). Additional comparisons of the gut microbiotas of different age classes (adult, juvenile) and sexes (male, female) are needed to clarify. For CS samples, CS1.1 had a higher relative abundance of Firmicutes (97.89 vs. 58.85%) and lower relative abundance of Bacteroidetes (0.90 vs. 32.95%). These results suggest that this individual ate a higher proportion of plant-based foods, but this difference is likely not due to age class, sex, and size. Although the WS1.1 and CS1.1 were visually different from their groups, they were still statistically representative of wild and captive populations, respectively, and results from statistical analyses excluding these individuals were similar. All the findings above suggest that the gut microbiota of wild and captive *S. bealei* are different, with diet likely being a major influence.

### Identification of Candidate Genera

By comparing the prevalence of taxa in wild and captive individuals, we are able to identify potentially important components in the gut microbiome. The genera significantly higher in WS were *Citrobacter*, unclassified Burkholderiaceae, *Plesiomonas*, *Hafnia-Obesumbacterium*, *Acinetobacter*, and *Lactococcus* (Figure 5B), while significantly higher in CS were *Clostridium*, *Turicibacter*, *Terrisporobacter*, and unclassified Lachnospiraceae (Figure 5B). We explore the bacterial literature to try to understand the function of these candidate genera in the gut of *S. bealei* and identified three of genera that have potentially important functions. *Citrobacter* (phylum Proteobacteria) is represented by OTU404, whose species identification was uncertain, being either *Ci. freundii*, *Ci. werkmanii*, or *Ci. portucalensis* (Supplementary Figure S4 and Supplementary Table S5). Generally, *Citrobacter* can use citrate (a derivative of citric acid) as a sole carbon source. Citric acid is common in some fruits, and this could indicate the importance of fruits in the diet of wild *S. bealei*. This inference aligns with a visual fecal content analysis of *S. bealei*, which found that fecal samples of over 87% of individuals studied contained fruits and seeds of six native species (*Celtis* sp., *Diospyros* sp., *Elaeocarpus* sp., *Garcinia oblongifolia*, *Ilex* sp., *Machilus chekiangensis*, and *Microcos nervosa*; Sung et al., 2020).

*Clostridium* (phylum Firmicutes) represented by 14 OTUs (Supplementary Figure S4 and Supplementary Table S5) was more common in CS individuals, and this genus of bacteria is common in the gut of humans (Suau et al., 1999) and fish (Kim et al., 2007; Wu et al., 2012; Burgos et al., 2018). One of its major functions is to help a host digest plant-based foods by fermenting cellulose (Burgos et al., 2018). This matches with our finding that captive individuals have a plant-rich diet. Lastly, *Terrisporobacter* (phylum Firmicutes) is represented by OTU8, which was identified to be *T. glycolicus/mayombei* (Supplementary Figure S4 and Supplementary Table S5). *Terrisporobacter glycolicus/mayombei* has been identified as an emerging anaerobic pathogen (Cheng et al., 2016). Although we cannot evaluate the pathogenicity of this OTU, the presence of this OTU may be an indicator of poor health of captive *S. bealei*. Additional studies clarifying the function of these candidate taxa, by both functional analyses and isolation and testing of strains, will useful in helping us understand their role in the gut of *S. bealei*.

### Turtle Gut Microbiota

Studies of turtle gut microbial diversity have found influences of diet (Modica, 2016; Bloodgood et al., 2020), geography (Butterfield, 2019), and ontogeny (Yuan et al., 2015; Peng et al., 2020). For the influence of diet, the pattern in turtles was similar to that seen in humans; individuals with plant-based diets had gut microbiota dominated by Firmicutes, while individuals with animal-based diets had gut microbiota dominated by Bacteroidetes (David et al., 2014). Gut microbiota dominated by Firmicutes were found in herbivorous tortoises (Gaillard, 2014; Modica, 2016) and sea turtles (Abdelrhman et al., 2016; Biagi et al., 2018; Arizza et al., 2019; Bloodgood et al., 2020). Our study provides some of the first data on the taxonomic composition of *S. bealei* gut microbiome. The gut microbiota of wild *S. bealei* is dominated by Proteobacteria, a phylum of bacteria connected to a variety of metabolic functions (Moon et al., 2018). We suggest that a Proteobacteria-dominant gut microbial community is more resilient and adaptable to the changing environment in nature. This may also be connected to the more diverse diet in the wild, which for *S. bealei* includes both plant and animal items and dominated by fruit and terrestrial insects (Sung et al., 2020).

The gut microbiota work on freshwater turtles has only covered a few North American species (family Emydidae) (Butterfield, 2019; Fugate et al., 2019; Peng et al., 2020). Our work is the first on an Asian freshwater turtle species (family Geoemydidae). Our results for wild *S. bealei* at the phylum level was similar to one species (*Graptemys psesudogeographica*) (Butterfield, 2019) dominated by Proteobacteria, while different from other species (*Chrysemys picta* and *Trachemys scripta elegans*) (Fugate et al., 2019; Peng et al., 2020) dominated by Firmicutes. The limited amount of gut microbiota data for turtles and especially freshwater turtles prevents us from making any generalizations, but we hope that future studies will help us understand the host-microbiota relationship in turtles.

### Importance for Turtle Conservation

Although a lot remains unknown about the host-microbiota relationship in turtles, these findings can be used to improve the management of the captive breeding colony of *S. bealei*. It has always been a best practice to provide a diet that mimics that in nature, and our study suggests how the health of captive individuals can be affected. We identify taxa that are potentially beneficial (*Cetobacterium*, *Citrobacter*) and expand our understanding of how diet influences gut microbiota diversity.

Compared to wild individuals, captive individuals likely had a higher plant-based diet indicated by higher levels of Firmicutes (especially *Clostridium*), and also had a deficiency in fruit in their diet as seen by the absence of *Citrobacter*. What superficially seems like a contradiction may indicate an important difference in how we classify food items. Fruit are part of a plant, but differ in chemical and nutritional content to vegetative parts. The plant-based ingredients in the commercial food (soybean meal, wheat flour) would be digested, absorbed, and incorporated differently than fruit. Based on our results, we suggest that the health of captive *S. bealei* could be improved by including more fruit in the diet, with a preference for native fruits when possible (*Celtis* sp., *Diospyros* sp., *Elaeocarpus* sp., *Garcinia oblongifolia*, *Ilex* sp., *Machilus chekiangensis*, and *Microcos nervosa*; Sung et al., 2020). We suggest that this in turn may improve the reproductive success in captivity.

## Conclusion

In this study, we characterize and compare the fecal microbiota of wild and captive *S. bealei*. Although our sample size was relatively large for endangered Asian turtles, the sample size in a statistical sense are small, and therefore our findings should be considered tentative. This is the first characterization of fecal microbiota for an Asian freshwater turtle. The results will help in the conservation of an endangered turtles and provide scientific basis to improve the management of the captive breeding colony. We highlight studies of fecal microbiota as a tool in assisting the management of endangered turtles (e.g., captive breeding colonies), such as modifying diet to mimic natural conditions, identifying potentially important bacterial taxa (*Cetobacterium*, *Citrobacter*, *Clostridium, Terrisporobacter*).

## Data Availability Statement

The datasets generated for this study can be found in the online repositories. The names of the repository/repositories and accession number(s) can be found in the article.

## Ethics Statement

The animal study was reviewed and approved by the Lingnan University Research Ethics Committee. Written informed consent was obtained from the owners for the participation of their animals in this study.

## Author Contributions

JJF, Y-HS, and LD designed the study and wrote the manuscript. Y-HS collected the samples. JJF and LD analyzed the data. All authors contributed to the article and approved the submitted version.

## Conflict of Interest

The authors declare that the research was conducted in the absence of any commercial or financial relationships that could be construed as a potential conflict of interest.

## Footnotes

1 http://www.arb-silva.de

## Supplementary Material

Supplementary Figure 1 Photographs of device used to collect fecal samples. **(A)** Overview of the device with space for four individuals. **(B)** Closeup of turtle in container, with the mesh floor. Once an individual excretes feces, it falls down onto the collecting tray (sterilized with 10% bleach) and is collected immediately.

Supplementary Figure 2 The length distribution of valid sequences.

Supplementary Figure 3 Rarefaction curves of the samples.

Supplementary Figure 4 Phylogeny identifying candidate genera to species. This tree is based on a maximum likelihood analysis performed in RAxML. Numbers at nodes represent bootstrap values and are shown only if >70. OTUs to be identified have bold font (WS, wild *Sacalia bealei*; CS, captive *Sacalia bealei*). Other taxa in the phylogeny were identified using BLAST and downloaded from GenBank. Accession numbers follow the species names.

Supplementary Table 1 General information for each sample. WS, Wild *Sacalia bealei*; CS, Captive *S. bealei*. The first number in the name indicates the locality collected, while the second number indicates the individual. Captive individuals are considered to be from one locality since they have been in captivity for approximately seven years (since 2013).

Supplementary Table 2 The results of statistical analysis for bacteria relative abundance at the phylum level for wild (WS) and captive (CS) *Sacalia bealei*. Phyla showing significant difference between WS and CS are in bold font.

Supplementary Table 3 The results of statistical analysis for bacteria relative abundance at the family level for wild (WS) and captive (CS) *Sacalia bealei*. Families showing significant difference between WS and CS are in bold font.

Supplementary Table 4 The results of statistical analysis for bacteria relative abundance at the genus level for wild (WS) and captive (CS) *Sacalia bealei*. Genera showing significant difference between WS and CS are in bold font.

Supplementary Table 5 Identification of candidate genera to species. A maximum of five BLAST results are included. WS, wild *Sacalia bealei*; CS, captive *Sacalia bealei*.
