# Supplementary figures and images for: Comparative Analysis of the Fecal Microbiota of Wild and Captive Beal’s Eyed Turtle (Sacalia bealei) by 16S rRNA Gene Sequencing
Source: Front Microbiol. 2020 Nov 6;11:570890. doi: 10.3389/fmicb.2020.570890 (PMC7677423; doi:10.3389/fmicb.2020.570890)

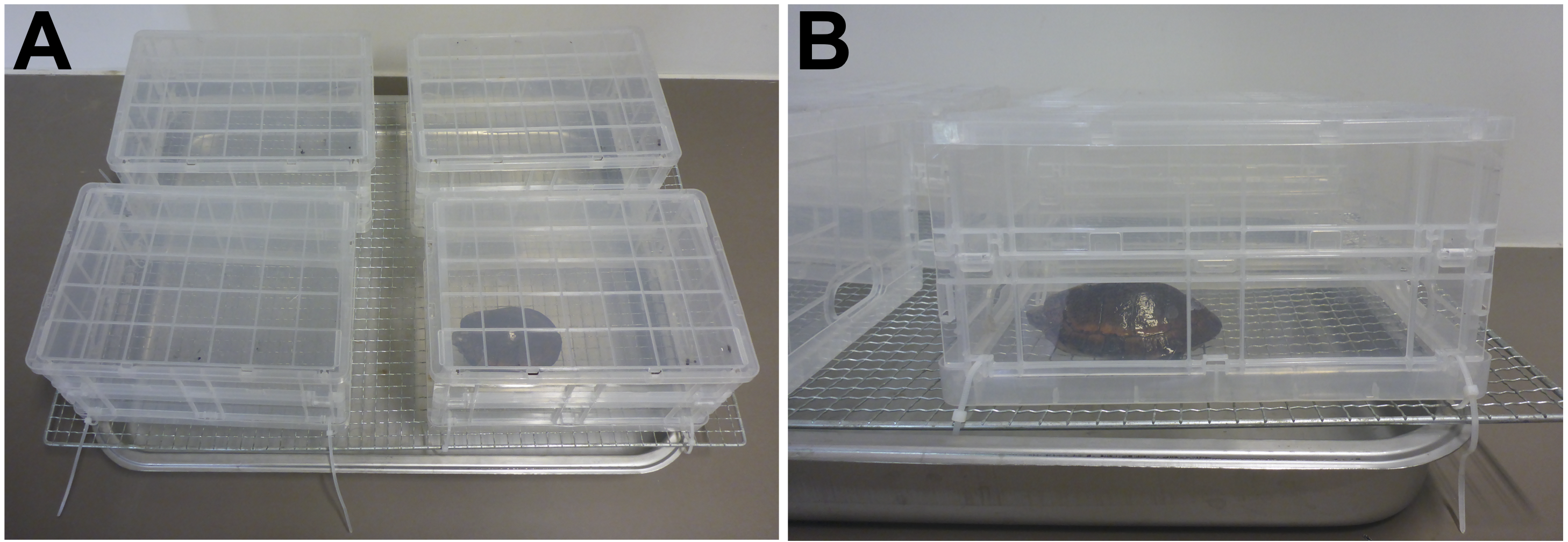

Supplement: Supplementary Figure 1 — Photographs of device used to collect fecal samples. (A) Overview of the device with space for four individuals. (B) Closeup of turtle in container, with the mesh floor. Once an individual excretes feces, it falls down onto the collecting tray (sterilized with 10% bleach) and is collected immediately. [file Image_1.tiff]

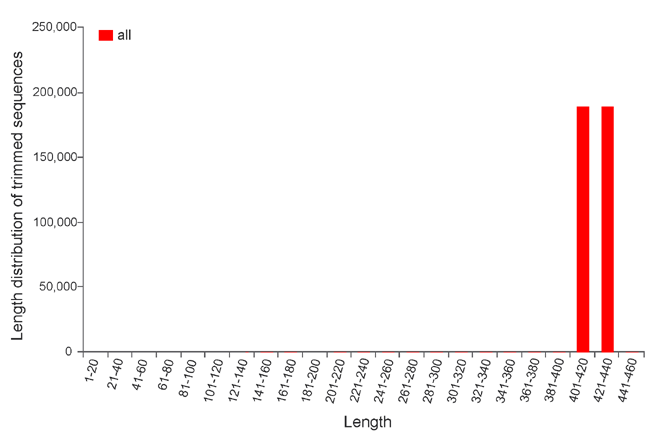

Supplement: Supplementary Figure 2 — The length distribution of valid sequences. [file Image_2.TIFF]
