# Supplementary material for: Comparative Analysis of the Fecal Microbiota of Wild and Captive Beal’s Eyed Turtle (Sacalia bealei) by 16S rRNA Gene Sequencing
Source: Front Microbiol. 2020 Nov 6;11:570890. doi: 10.3389/fmicb.2020.570890 (PMC7677423; doi:10.3389/fmicb.2020.570890)
Supplement: Supplementary Table 1 — General information for each sample. WS, Wild Sacalia bealei; CS, Captive S. bealei. The first number in the name indicates the locality collected, while the second number indicates the individual. Captive individuals are considered to be from one locality since they have been in captivity for approximately seven years (since 2013). [file Table_1.DOCX]

**Table S1** General information for each sample. WS=Wild *Sacalia bealei*, CS=Captive *S. bealei*. The first number in the name indicates the locality collected, while the second number indicates the individual. Captive individuals are considered to be from one locality since they have been in captivity for approximately seven years (since 2013).

| **Sample** | **Age class & sex** | **Sequencing** | | | | | |
| --- | --- | --- | --- | --- | --- | --- | --- |
|  |  | **Carapace length (mm)** | **Number of reads** | **Number of bases** | **Mean length** | **Min length** | **Max length** |
| WS1.1 | Juvenile | 58.5 | 50,552 | 21,100,822 | 417.41 | 249 | 432 |
| WS1.2 | Adult female | 111.0 | 46,793 | 19,787,695 | 422.88 | 262 | 432 |
| WS1.3 | Adult female | 120.1 | 62,393 | 25,821,244 | 413.85 | 261 | 431 |
| WS2.1 | Adult female | 143.3 | 54,102 | 22,669,203 | 419.01 | 400 | 433 |
| CS1.1 | Adult male | 143.0 | 32,115 | 13,303,526 | 414.25 | 252 | 431 |
| CS1.2 | Adult female | 148.0 | 71,094 | 29,423,257 | 413.86 | 252 | 435 |
| CS1.3 | Adult male | 113.0 | 61,577 | 25,543,610 | 414.82 | 252 | 431 |
| CS1.4 | Adult female | 152.0 | 53,632 | 22,082,997 | 411.75 | 252 | 444 |
