# Supplementary material for: Comparative Analysis of the Fecal Microbiota of Wild and Captive Beal’s Eyed Turtle (Sacalia bealei) by 16S rRNA Gene Sequencing
Source: Front Microbiol. 2020 Nov 6;11:570890. doi: 10.3389/fmicb.2020.570890 (PMC7677423; doi:10.3389/fmicb.2020.570890)
Supplement: Supplementary Table 3 — The results of statistical analysis for bacteria relative abundance at the family level for wild (WS) and captive (CS) Sacalia bealei. Families showing significant difference between WS and CS are in bold font. [file Table_3.docx]

**Table S3** The results of statistical analysis for bacteria relative abundance at the family level for wild (WS) and captive (CS) *Sacalia bealei*. Families showing significant difference between WS and CS are in bold font.

| **sample** | WS1.1 | WS1.2 | WS1.3 | WS2.1 | CS1.1 | CS1.2 | CS1.3 | CS1.4 | Kolmogorov-Smirnov test *P* value | Homogeneity-Variances test  *P* value | Student’s t-test  *P* value | Wilcoxon rank-sum test  *P* value |
| --- | --- | --- | --- | --- | --- | --- | --- | --- | --- | --- | --- | --- |
| unclassified Bacteroidales | 0 | 2.030603407 | 25.17884066 | 18.16636192 | 0.198890065 | 33.77602412 | 44.99727328 | 17.77499759 | 0.2 | 0.322 | 0.3079 |  |
| **Clostridiaceae** | **0.086613415** | **2.207038142** | **11.44580246** | **9.883553075** | **21.84582812** | **10.67911334** | **25.16280114** | **24.16193501** | **0.2** | **0.945** | **0.01554** |  |
| **Enterobacteriaceae** | **2.608026177** | **54.42209604** | **6.896994194** | **29.28495814** | **0.009623713** | **0.660828281** | **0.048118564** | **0.012831617** | **0.005** | **0.013** |  | **0.03038** |
| Peptostreptococcaceae | 0.381740609 | 0.497225163 | 7.77916787 | 9.854681936 | 33.07670099 | 6.072562795 | 8.824944664 | 15.71552305 | 0.1 | 0.26 | 0.1352 |  |
| **Erysipelotrichaceae** | **0** | **0.012831617** | **0.102652937** | **0.86292625** | **41.0355115** | **5.73894075** | **7.33647708** | **10.21396722** | **0.018** | **0.029** |  | **0.03038** |
| Fusobacteriaceae | 0 | 2.10E+01 | 33.71507394 | 3.01543002 | 0.381740609 | 5.665158952 | 0.035286947 | 0.455522407 | 0.013 | 0.008 |  | 0.4705 |
| Lachnospiraceae | 0 | 0.782728643 | 2.94806403 | 7.682930741 | 1.167677157 | 19.77031405 | 5.212844449 | 11.22445706 | 0.2 | 0.139 | 0.1903 |  |
| Burkholderiaceae | 31.3925512 | 0.670451994 | 0.035286947 | 5.674782664 | 0.032079043 | 0.099445 | 0.009623713 | 0.016039521 | 0.001 | 0.030 |  | 0.0606 |
| Sphingomonadaceae | 16.80941841 | 0.686491515 | 0 | 0.132 | 0 | 0 | 0.006415809 | 0.003207904 | 0 | 0.024 |  | 0.1832 |
| **Ruminococcaceae** | **0** | **0.025663234** | **0.150771501** | **1.50771501** | **0.455522407** | **5.058865043** | **3.910435313** | **5.918583389** | **0.143** | **0.163** | **0.03444** |  |
| **Moraxellaceae** | **0.619125525** | **10.5764604** | **1.584704712** | **0.11227665** | **0** | **0** | **0.003207904** | **0** | **0.001** | **0.027** |  | **0.02652** |
| Acidaminococcaceae | 0 | 0.006415809 | 0 | 2.611234081 | 0.02245533 | 0.654412472 | 0.012831617 | 7.971642126 | 0.003 | 0.111 |  | 0.1913 |
| **Streptococcaceae** | **6.216918487** | **4.019504058** | **0.012831617** | **0.3753248** | **0** | **0** | **0** | **0** | **0** | **0.002** |  | **0.02107** |
| Rhizobiales *incertae sedis* | 8.693420588 | 0.016039521 | 0 | 0 | 0 | 0 | 0 | 0 | 0 | 0.024 |  | 0.1859 |
| Pseudomonadaceae | 5.944246624 | 0.105860841 | 0.102652937 | 1.976069034 | 0.317582523 | 0.038494851 | 0.051326468 | 0.019247426 | 0.001 | 0.057 |  | 0.1124 |
| Hyphomicrobiaceae | 6.768678023 | 0 | 0 | 0.003207904 | 0 | 0 | 0 | 0 | 0 | 0.024 |  | 1 |

Note: All data were tested for the normality and uniformity using Kolmogorov-Smirnov Test (K-S) and Homogeneity Variances Test (H-V), respectively. If the *P* value of K-S and H-V tests were greater than 0.05, statistical analysis was performed using Student’s t-test. If the *P* value of K-S and H-V tests were less than 0.05, statistical analysis was performed using Wilcoxon rank-sum test.
